# On QSAR modeling with novel degree-based indices and thermodynamics properties of eye infection therapeutics

**DOI:** 10.3389/fchem.2024.1383206

**Published:** 2024-05-27

**Authors:** Muhammad Waheed Rasheed, Abid Mahboob, Iqra Hanif

**Affiliations:** Department of Mathematics, Division of Science and Technology, University of Education, Lahore, Pakistan

**Keywords:** pharmaceutical chemistry, first revised randic index, second revised randic index, vertex degrees, QSPR analysis, linear regression model

## Abstract

Topological descriptors are numerical results generated from the structure of a chemical graph that are useful in identifying the physicochemical characteristics of a wide range of drugs. The introduction of molecular descriptors advances quantitative structure-property relationship research. This article focuses on the nine degree-based topological indices and the linear regression model of the eye infection drugs. We introduced two new indices, namely, the “first revised Randic index” and the ”second revised Randic index, for the analysis of eye infection drugs. Topological indices are calculated by using edge partitioning, vertex degree counting, and vertex degree labeling. This analysis is done with a scientific calculator and then authenticated with Matlab, a potent tool for examining data. The experimental data and results of the topological indices serve as inputs for the statistical computations and provide the values of intercepts, slopes, and correlation coefficients. All the correlations for the eye-infection drugs are positive, indicating a direct relationship between the experimental and estimated results of the drugs. There are significant results of the p-test for all of the characteristics of eye infection, such as molecular weight, boiling point, enthalpy, flash point, molar refraction, and molar volume, that validate the accuracy of the computations. A significant link was determined in this study between the defined indices with two properties: molar weight and molar refraction. The molar weight and molar refraction have a correlation coefficient ranging from 0.9. These results demonstrate a strong association between the indices and the properties under investigation. The linear regression approach is a valuable tool for chemists and pharmacists to obtain data about different medicines quickly and cost-effectively.

## 1 Introduction

An eye infection is a disorder brought on by organisms such as viruses, fungi, or bacteria. Eye infections are common in particular areas of the eye: the eyelid, cornea, and conjunctiva. There are several types of eye infections, including conjunctivitis, styes, keratitis, cellulitis, blepharitis, uveitis, ocular herpes, and dacryocystitis. Conjunctivitis, commonly known as “pink eye” occurs when a virus or bacterium infects the vessels in the conjunctiva. This highly contagious condition can spread quickly and easily, making it a formidable foe. A common infection known as a stye can cause a painful, red lump to form on the edge of the eyelid. Styes occur when an oil gland on the eyelid is blocked, resulting in a minor but uncomfortable bacterial infection. Fortunately, most cases of stye can be treated with simple home remedies (Watson et al., 2018). The cornea can become infected by bacteria, viruses, or fungi, resulting in keratitis. Additionally, eye injuries can also lead to this condition. Those who wear contact lenses are particularly susceptible to keratitis, making them more vulnerable to this infection. The tear ducts can become blocked due to dacryocystitis, which can be acquired through a bacterial infection or present at birth.

The symptoms of an eye infection can vary depending on the underlying cause and may include redness, irritation, watery eyes, light sensitivity, puffiness, discharge, dryness, itching, swelling around the eyes, and blurred vision. In some cases, these symptoms can be indicative of a more serious condition, so it is important to seek medical attention if any of these symptoms are present. The majority of common bacterial eye infections can be cured with prompt treatment, which typically involves the use of eye compresses, prescription antibiotic ointments, and prescription antibiotic eye drops. These infections can be effectively controlled and prevented from developing into further complications by acting quickly. It is essential to abstain from touching infected eyes, wash your hands thoroughly when handling contact lenses, and bear in mind that sleeping while wearing contacts may be detrimental to your ocular health (Doan and Pinsky, 2016).

In this manuscript, the physicochemical properties of the drugs used for the treatment of eye infections are analyzed using degree-related topological indices. Chemical graph theory is utilized to get information from chemical structures regarding eye infection medications. Chemical graph theory is the study of chemical structures with the help of different mathematical techniques, especially molecular descriptors. The molecular descriptor is the final outcome of a logical and mathematical process that translates the chemical information encoded in a molecule’s symbolic representation into a meaningful numerical value, or the result of a standardized experiment. A topological index is a particular type of molecular descriptor in two dimensions that discusses size, degree of branching, flexibility, and the neighborhood of atoms. A topological index is a function *f*: *G* → *N* that assigns a numerical value to each graph of chemical structures, making it a unique graph invariant due to its ability to estimate the physicochemical parameters of chemical compounds. Its usefulness has made it a valuable tool for chemists (Gnanaraj et al., 2023). Quantitative structure-activity and structure-property relationships (QSAR) require representations of the topological features of chemical structures. QSAR utilizes chemical graph theory to describe hydrogen-exhausted graphs of organic chemical networks, with the vertices and edges of the unique chemical components bonds corresponding to the points. By leveraging this approach, researchers can gain a better understanding of the relationship between the structure of a molecule and its activity or property. Chemical network theory is a branch of applied mathematics that attempts to model the behavior of real-world chemical systems.

A topological index should have a structural interpretation, demonstrate a good correlation with at least one property, discriminate among isomers, be locally defined, not be trivially related to other descriptors, be generalizable to higher analogues, be simple, not be based on properties, be possible to construct efficiently, be based on familiar structural concepts, show a correct size dependence, and change gradually with changes in structures. For more information about the topological indices, see (Mahboob et al., 2021; Lee et al., 2022a).

The concept of a topological index was proposed by Harry Wiener for approximating the boiling points of alkanes (Wiener, 1947). The Wiener index was a distance-related index called “path number” by him and counted the shortest distance between any two vertices of a graph. The degree-related T-indices are used in this article. The first-degree related T-indices are Zagreb indices used to calculate the pi-electron energy of the hydrocarbons. The Randic index is also a degree-related, famous index introduced for the analysis of branching structures in hydrocarbons.The main purposes of this manuscript are:

• The QSAR analysis of the structures of eye infection drugs with the help of molecular descriptors
• Predict the properties of eye infection medicines using a theoretical approach without expensive and time-consuming experiments
• Calculation of the values of topological indices
• To calculate the linear regression and correlation coefficients for comparison of experimental and estimated data
• The results and their comparisons are analyzed and discussed in a graphical way. The line graphs and bar graphs are suitable for demonstrating the simple statistical parameters.

The medicines for the eye infection are examined, evaluated, and estimated with the help of molecular descriptors. A large number of articles with useful applications are published on the same method for different drugs. These articles motivated us to work on regression analysis and degree-related indices. Tharmalingam et al. applied the degree-related T-indices to study the hexabenzocorenene structures (Tharmalingam et al., 2023). He studied the vertex degree-related and bond additive descriptors to generalize the benzene structures. Saima et al. used simple linear regression and nine degree-related descriptors to understand the properties of Vitiligo disease (Parveen et al., 2022a). The QSPR model outcomes for Vitiligo disease demonstrate that the ABC index has a strong correlation with molar volume (r = 0.858), while the F index has the highest associated value of complexity (r = 0.952). Moreover, the M index exhibits the highest correlation coefficient of refractivity (r = 0.971), and the H-index has the highest correlated value of enthalpy (r = 0.838). A new index called the Albertson index is applicable for the determination of polarizability with values of correlation r = 0.571. Abid et al. recently applied the degree-related indices and different regression models on the Lyme disease (Huang et al., 2023). Cardiovascular disorder is a heart-related disease. Bashir et al. studied the characteristics of the heart disease structures such as boiling point, flash point, enthalpy, molar refraction, polarity, complexity, density, and surface area (Bashir et al., 2022). It was observed that all these properties have a strong relationship with the calculation of molecular descriptors. Zhang theoretically predicted the properties of the malaria drugs with the help of a linear regression model and a graphical approach (Zhang et al., 2023). Shanmukha et al. conducted a QSPR analysis of 17 anti-cancer medicines, calculating 13 topological indices to assess the correlation between the drugs and five physicochemical parameters. The results of the regression analysis revealed a significant positive relationship between all five parameters (Shanmukha et al., 2020). For more information about the different types of degree-related indices and the analysis of different drugs with the help of regression models, see (Kirmani et al., 2021; Parveen et al., 2022b).

The degree-based topological indices used in this article give significant results for estimating the properties of the eye-infection drugs. The topological indices are the numerical quantities that describe information about different chemical structures by theoretical method. Vertex degree-based indices are a structural analysis tool that focuses on individual atoms within a molecule, highlighting their connectivity patterns. Higher degrees indicate more bonds, suggesting higher levels of branching or substitution. These indices can also reflect the level of branching or complexity within a molecule, often corresponding to branching points or functional groups. The degree of an atom can influence its reactivity and chemical behavior, with higher degrees allowing for greater accessibility to chemical reactions. The connectivity of atoms can also affect the steric hindrance and spatial arrangement of substituents, affecting the overall molecular conformation and stability. Additionally, vertex degree-based indices can provide insights into the distribution of electrons within a molecule, particularly in conjugated systems, influencing the molecular electronic structure and properties.

There are eight main sections to this article, each of which covers a different aspect of the topic. Section 1 provides definitions, information, motivation, and a literature review of the formulae. Section 2 discusses the techniques and methods used for the computation of results. Section 3 covers the fundamentals of the chemical structures used in eye infection drugs as well as the experimental values of the characteristics and outcome of T-indices. Section 4 examines the linear regression and correlation coefficients between the properties of drugs and T-indices. Section 5 takes a graphical approach to the representation of data and studies the results related to recent work on linear regression and different diseases. Section 6 presents the application of the T-indices used in this article. Finally, Section 7 offers a discussion. Section 8 consist of concluding remarks, and suggestions for future work. All references to the topics discussed in the article are included last.

### 1.1 Fundamental definitions and literature review

Suppose G = (E, V) is a graph representing the two-dimensional structures of eye infection medicines. In graphs, there are two basic sets: the edge set E(G) = {*e*_1_, *e*_2_, *e*_3_, …, *e*_*n*_} and the vertex set V(G)= {*a*, *b*, …, *z*}. All the graphs are two-dimensional, planar, and without loops. The degree of a vertex g is the number of edges attached to the vertex g, represented as Φ_*g*_. Two vertices are adjacent if there is a common edge between vertices. The general formula for the degree-related topological indices is given below.The mathematical definition of the topological indices is:

$$TI\left(G\right)=\underset{\left\{g,h\right\}\subseteq V\left(G\right)}{\sum }F\left(\Phi \left(g\right),\Phi \left(h\right)\right),$$ (1.1)

where Φ(*g*) and Φ(*h*) are the inputs, and F is a function.In 1972, Gutman and Trinajstic introduced a formula to investigate the effect of total pi-electron energy on a molecule’s structure called the Zagreb indices (Gutman and Trinajstic, 1972). Nikolic et al. studied the Zagreb indices after 30 years and gave all the possible information about the progress of the Zagreb index (Nikolic et al., 2003). They analyzed the different models used for estimating the properties of the alkanes. Due to the great success of Zagreb indices, different modified and advanced forms with different approaches are calculated for many simple as well as complicated graphs. These indices are applied to both organic and inorganic compounds. The Zagreb indices are defined as:

$$M_{1}\left(G\right)=\underset{gh\varepsilon E\left(G\right)}{\sum }\left(\Phi_{g}+\Phi_{h}\right),$$

$$M_{2}\left(G\right)=\underset{gh\varepsilon E\left(G\right)}{\sum }\left(\Phi_{g}\times \Phi_{h}\right).$$

In a recent study, Adnan et al. discovered that the Z-indices are useful for calculating the physical and chemical properties of anti-tuberculosis drugs (Hao, 2011; Adnan et al., 2022). This results highlights the versatility and practicality of the Zagreb indices in various fields of research.In 2003, Nikolic et al. introduced the three modified forms of the Zagreb indices for the analysis of the boiling point of alkanes (Nikolic et al., 2003). The modified indices have proved to be very useful for some molecular structures, but not too much work is done on this index. The modified indices are studied and discussed collectively to analyze the different molecular structures. The second modified index is abbreviated as ^*m*^ *M*_2_(*G*) and defined as follows:

M2mG=∑ghεEG1Φg×Φh.

In 1987, Siemion Fajtlowicz introduced a formula for understanding complex computer networks called the “harmonic index,” (Fajtlowicz, 1987). Most of the indices are introduced by chemists to be helpful in chemistry and pharmacology, but the harmonic index is one of the indices calculated while studying computer networks. In 2012, Zhong introduced the same formula individually for determining the properties of trees, especially for alkanes. He gave the name “Harmonic index” to this formula (Zhong, 2012). The mathematical definition of H-index is as follows:

$$H\left(G\right)=\underset{gh\varepsilon E\left(G\right)}{\sum }\frac{2}{\left(\Phi_{g}+\Phi_{h}\right)}.$$

In 2015, Gutman and Furtula proposed the forgotten index for the prediction of the properties of isomers of octane (Furtula and Gutman, 2015). This index was discussed in the same article where Zagreb indices were introduced in 1972, but no one gave importance to this index. So after 43 years, Gutman and Furtula noticed that the forgotten index is a very efficient and powerful index for calculating the characteristics of organic and inorganic compounds. Bokhary et al. applied the harmonic index to predict the characteristics of the breast cancer medicines (Bokhary et al., 2022) and observed that F(G) was highly correlated with the molar volume of the breast cancer drugs with a correlation coefficient of 0.931 at the molecular level. The forgotten index is represented by F(G) and defined as follows:

$$F\left(G\right)=\underset{gh\varepsilon E\left(G\right)}{\sum }\left(\Phi_{g}^{2}+\Phi_{h}^{2}\right).$$

In 2013, Shirdel et al. introduced the hyper Zagreb index for the cartesian product, disjunction, composition, and join of the simple graphs (Shirdel et al., 2013). Anil et al. increase the worth of the HM index by applying this index for the prediction of asthma drugs (Anil et al., 2020). The HM index is highly correlated with the boiling point, with a correlation value of 0.954. The hyper zagreb index is denoted by HM and defined as follows:

$$HM\left(G\right)=\underset{gh\varepsilon E\left(G\right)}{\sum }{\left(\Phi_{g}+\Phi_{h}\right)}^{2}.$$

In 2010, Vukicevic and Gasperov proposed the symmetric division index (Vukicevic and Gasperov, 2010). They studied the 148 additive types of indices and gave a comparison of all the indices. The additive indices are divided into three classes: extended adriatic descriptors, variable adriatic descriptors, and discrete adriatic descriptors. Various indices show a good correlation with the properties of compounds, but some indices did not correlate with any compound. The symmetric division index is a very useful and beneficial index. In 2020, after 10 years, Ali et al. discussed the symmetric division index for numerous molecular graphs (Ali et al., 2020). He discussed the upper and lower bounds of the SDD index for trees and path graphs. The symmetric division index is abbreviated as SDD and defined as follows:

$$SD\left(G\right)=\underset{gh\varepsilon E\left(G\right)}{\sum }\left(\frac{\Phi_{g}}{\Phi_{h}}+\frac{\Phi_{h}}{\Phi_{g}}\right)$$

In this article, we have defined two new topological indices by inspiring the Randic index, called the first revised Randic index (FRR) and the second revised Randic index (SRR). The first revised Randic index is defined as:

$$FRR\left(G\right)=\underset{gh\varepsilon E\left(G\right)}{\sum }\frac{1}{\sqrt{\Phi_{g}\times \Phi_{h}}+\sqrt{\Phi_{g}+\Phi_{h}}}.$$

The mathematical definition of the second revised Randic index is given below. The term Φ(*h*) stands for the higher degree vertex, and Φ(*l*) is the lower degree vertex in an edge *gh*. The SRR-index deals with the concept of a higher and lower degree of vertex in an edge. It is formed by the division of the difference between higher and lower degrees by the square root of the multiplication of Φ(*h*) and Φ(*l*). The SRR index shows a very good correlation with the six properties of eye infection drugs.

$$SRR\left(G\right)=\underset{gh\varepsilon E\left(G\right)}{\sum }\frac{\Phi \left(h\right)-\Phi \left(l\right)+1}{\sqrt{\Phi \left(h\right)\times \Phi \left(l\right)}}$$

## 2 Techniques used for computations of results

**FIGURE 1:**
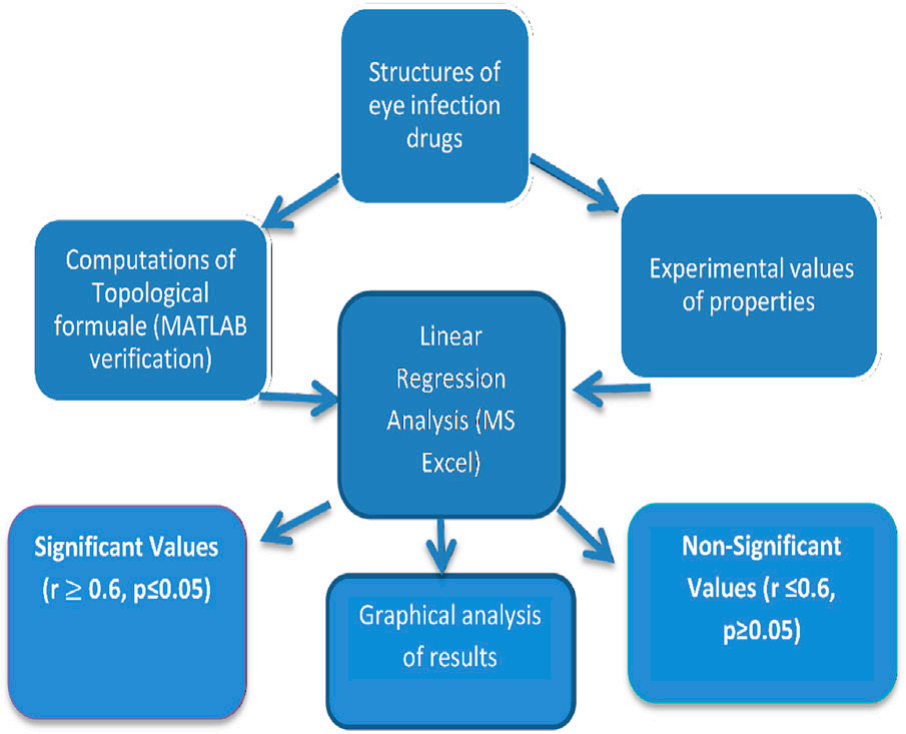
Procedure for computation of results.

This article discusses two types of calculations that are essential in pharmaceutical research: topological indices and statistical analysis. Topological indices are determined through edge partitioning, vertex degree counting, and vertex degree labeling. These calculations are performed using a scientific calculator and verified by Matlab, which is a powerful software tool for data analysis. To gather experimental data on eye infection drugs, we collected information from ChemSpider and used a linear regression model to analyze both experimental and estimated data. Microsoft Excel is used for statistical computations, and line graphs are drawn using the same software for graphical comparisons of data. Figure 1 illustrates the step-by-step procedure for the estimation of the properties of eye infection drugs.

## 3 Basic results about the properties of eye infection drugs

This section presents the fundamental results of the topological indices. Nine degree-related topological indices are utilized to analyze 20 molecular structures of medicines that are commonly used to treat various eye infections. Although the structures of these medicines are multi-dimensional, the two-dimensional forms of the eye infection medicines are examined by T-indices. This manuscript analysis has yielded valuable insights into the topological properties of eye infection medicines. These results have significant implications for the production of more effective drugs for eye infections.

### 3.1 Experimental results about properties of drugs

**FIGURE 2:**
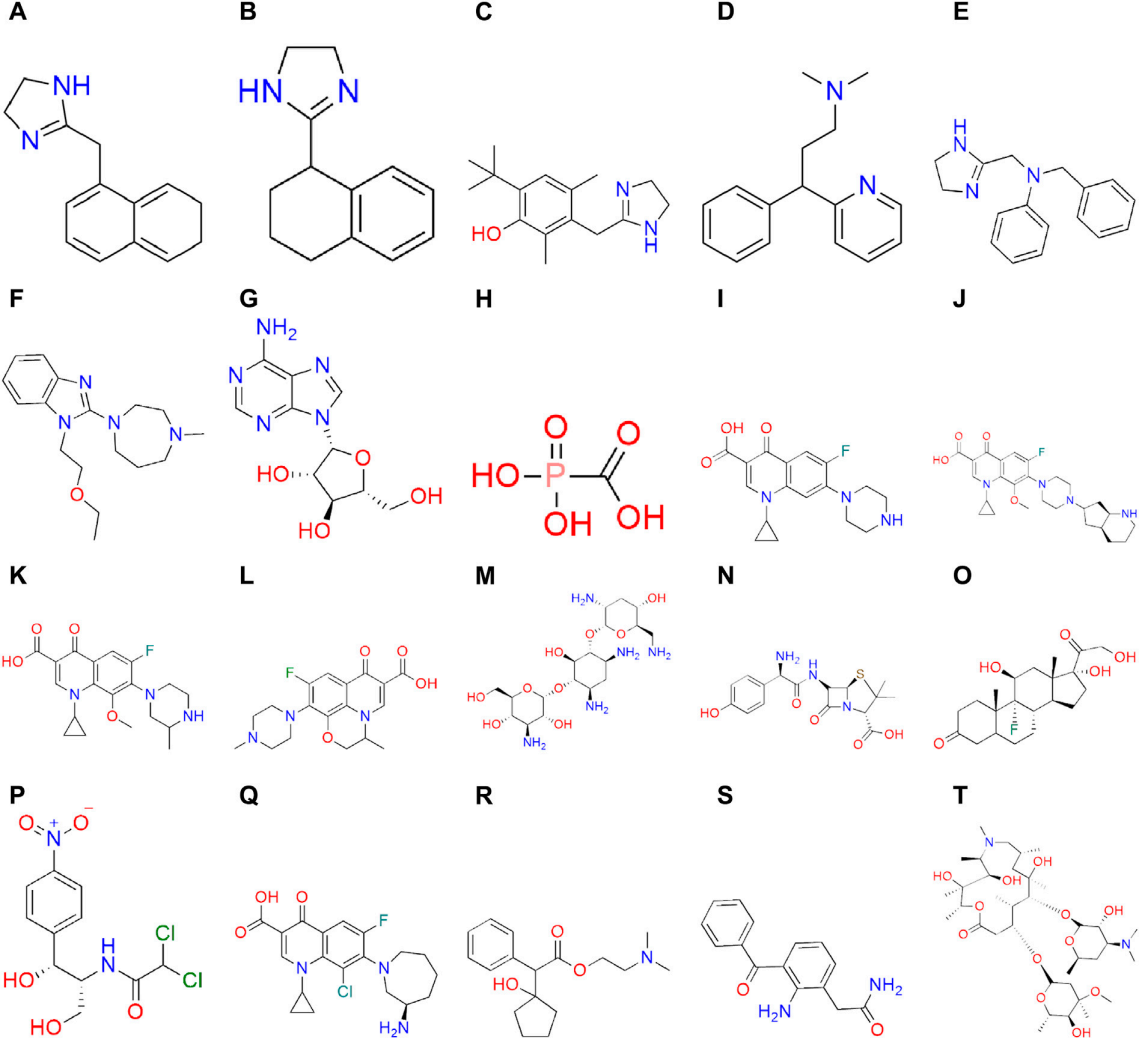
Structures of eyes infection medicines.

Six properties of eye infection drugs are estimated using numeric parameters. These properties are molar weight (MW), boiling point (BP), enthalpy of vaporization (EV), flash point (FP), molar refraction (MR), and molar volume (MV). The boiling point is the temperature at which a liquid transforms into a vapor, or when the vapor pressure is equal to the average atmospheric pressure at sea level. The boiling points in Table 1 are calculated in degrees Celsius and represent the amount of energy required to vaporize a given volume of a liquid material. The unit of EV in Table 1 is a kilojoule per mole, while the unit of FP is a degree Celsius. The data about MR and MV in this article is measured in cubic meters per mole. Table 1 shows that the experimental values of the parameters are not exact; they lie within a range. To calculate the correlation between two or more exact values, the exact values are used for the computations of the correlations and regression equations. All the structures and data about drugs are taken from ChemSpider, a reliable source for chemical information. The molecular structures of eye infection medicines are given in Figure 2.

**TABLE 1:** Physicochemical properties of eyes infection medicines.

Drug name	MW	BP	EV	FP	MR	MV
Naphazoline	210.274	440.5 ± 24	67.1 ± 3	220.2 ± 22.9	65.5 ± 0.5	181.8
Tetrahydrozoline	200.279	393.5 ± 21	61.8 ± 3	191.8 ± 22.1	60.9 ± 0.5	165.7
Oxymetazoline	260.375	431.9 ± 33	71.4 ± 3	215.0 ± 25.4	77.8 ± 0.5	240.9
Pheniramine	240.343	348.3 ± 37	59.3 ± 3	164.5 ± 26.5	75.9 ± 0.3	236.1
Antazoline	265.353	475.5 ± 38	73.9 ± 3	241.4 ± 26.8	83.3 ± 0.5	241.0
Emedastine	302.414	446.6 ± 55	70.5 ± 3	223.9 ± 31.5	89.2 ± 0.5	262.6
Vidarabine	267.241	676.3 ± 65	104.3 ± 3	362.8 ± 34.3	60.0 ± 0.5	128.2
Foscarnet	126.005	490.7 ± 28	82.9 ± 6	250.6 ± 24.0	18.2 ± 0.3	58.8
Ciprofloxacin	331.341	581.8 ± 50	91.5 ± 3	305.6 ± 30.1	83.3 ± 0.3	226.8
Moxifloxacin	401.431	636.4 ± 55	98.8 ± 3	338.7 ± 31.5	101.8 ± 0.3	285.0
Gatifloxacin	375.394	607.8 ± 55	95.0 ± 3	321.4 ± 31.5	94.6 ± 0.3	270.8
Ofloxacin	361.367	571.5 ± 50	90.1 ± 3	299.4 ± 30.1	91.1 ± 0.4	244.0
Tobramycin	467.514	775.4 ± 60	128.7 ± 6	422.8 ± 32.9	111.7 ± 0.4	305.9
Amoxicilin	365.404	743.2 ± 60	113.7 ± 3	403.3 ± 32.9	91.5 ± 0.4	236.2
Dexamethasone	392.461	568.2 ± 50	98 ± 6	297.5 ± 30.1	100.2 ± 0.4	296.2
Chloramphenicol	323.129	644.9 ± 55	100 ± 3	343.8 ± 31.5	72.6 ± 0.3	208.8
Besifloxacin	393.840	607.0 ± 55	94.9 ± 3	320.9 ± 31.5	97.6 ± 0.3	268.0
Cyclopentolate	291.385	409.6 ± 20	69.8 ± 3	201.5 ± 21.8	82.4 ± 0.3	256.5
Nepafenac	254.284	562.5 ± 50	84.6 ± 3	294.0 ± 30.1	73.4 ± 0.3	203.4
Azithromycin	748.984	822.1 ± 65	136 ± 6.0	451.0 ± 34.3	197.6 ± 0.4	632.7

### 3.2 Calculated results about properties of eye infection drugs

Nine degree-related topological indices, such as the first zagreb index, the second zagreb index, the modified second zagreb index, the harmonic index, the forgotten index, the hyper zagreb index, the symmetric division index, the first revised Randic index, and the second revised Randic index, are calculated for the structures of eye infection drugs. These indices are calculated using specific formulae and provide estimated or approximated values of the properties of medicines used to treat eye infections. While there are many topological indices that have been introduced to understand the characteristics of different graphs, these indices are particularly suitable for analyzing the structures of eye infection medicines. The maximum degree of the vertex is four, while the minimum degree is one. Additionally, there are nine parcels in the edge division of eye infection drugs. Different drug structures are divided into different edge parcels; for instance, the first structure given in Table 2 has only three edge divisions. Naphazoline, for example, has 18 edges in total, with the first edge packet *e*_1_ = (2, 2) = 8, the second edge packet *e*_2_ = (3, 2) = 8, and the third edge bundle *e*_3_ = (3, 3). Similarly, different structures have different edge divisions. All the indices are additive, and the hyper-zagreb index shows the highest values while the modified-second-zagreb index has the lowest values, as seen in Table 2. The possible edge bundles are given below.

$$\begin{array}{l} E_{\left(1,2\right)}=\left\{e=gh\epsilon E\left(G\right)|\Phi_{g}=1,\Phi_{h}=2\right\}\\ E_{\left(1,3\right)}=\left\{e=gh\epsilon E\left(G\right)|\Phi_{g}=1,\Phi_{h}=3\right\}\\ E_{\left(1,4\right)}=\left\{e=gh\epsilon E\left(G\right)|\Phi_{g}=1,\Phi_{h}=4\right\}\\ E_{\left(2,2\right)}=\left\{e=gh\epsilon E\left(G\right)|\Phi_{g}=2,\Phi_{h}=2\right\} \end{array}$$

$$\begin{array}{l} E_{\left(2,3\right)}=\left\{e=gh\epsilon E\left(G\right)|\Phi_{g}=2,\Phi_{h}=3\right\}\\ E_{\left(2,4\right)}=\left\{e=gh\epsilon E\left(G\right)|\Phi_{g}=2,\Phi_{h}=4\right\}\\ E_{\left(3,3\right)}=\left\{e=gh\epsilon E\left(G\right)|\Phi_{g}=3,\Phi_{h}=3\right\}\\ E_{\left(3,4\right)}=\left\{e=gh\epsilon E\left(G\right)|\Phi_{g}=3,\Phi_{h}=4\right\}\\ E_{\left(4,4\right)}=\left\{e=gh\epsilon E\left(G\right)|\Phi_{g}=4,\Phi_{h}=4\right\} \end{array}$$

$$M_{1}\left(Naphazoline\right)=\underset{gh\varepsilon E\left(G\right)}{\sum }\left(\Phi_{g}+\Phi_{h}\right)=8\left(2+2\right)+8\left(3+2\right)+2\left(3+3\right)=84$$

$$M_{2}\left(Naphazoline\right)=\underset{gh\varepsilon E\left(G\right)}{\sum }\left(\Phi_{g}\times \Phi_{h}\right)=8\left(2\times 2\right)+8\left(3\times 2\right)+2\left(3\times 3\right)=98$$

M2mNaphazoline=∑ghεEG1Φg×Φh=82+2+83+2+23+3=3.555

$$H\left(Naphazoline\right)=\underset{gh\varepsilon E\left(G\right)}{\sum }\frac{2}{\left(\Phi_{g}+\Phi_{h}\right)}=\frac{2\times 8}{2+2}+\frac{2\times 8}{3+2}+\frac{2\times 2}{3+3}=7.866$$

$$F\left(Naphazoline\right)=\underset{gh\varepsilon E\left(G\right)}{\sum }\left(\Phi_{g}^{2}+\Phi_{h}^{2}\right)=8\left(2^{2}+2^{2}\right)+8\left(2^{2}+3^{2}\right)+2\left(3^{2}+3^{2}\right)=204$$

$$HM\left(Naphazoline\right)=\underset{gh\varepsilon E\left(G\right)}{\sum }{\left(\Phi_{g}+\Phi_{h}\right)}^{2}=8{\left(2+2\right)}^{2}+8{\left(3+2\right)}^{2}+2{\left(3+3\right)}^{2}=400$$

$$SD\left(Naphazoline\right)=\underset{gh\varepsilon E\left(G\right)}{\sum }\left(\frac{\Phi_{g}}{\Phi_{h}}+\frac{\Phi_{h}}{\Phi_{g}}\right)=8\left(\frac{2}{2}+\frac{2}{2}\right)+8\left(\frac{3}{2}+\frac{2}{3}\right)+2\left(\frac{3}{3}+\frac{3}{3}\right)=37.33$$

$$\begin{array}{ll} FRR\left(Naphazoline\right) & =\underset{gh\varepsilon E\left(G\right)}{\sum }\frac{1}{\sqrt{\Phi_{g}\times \Phi_{h}}+\sqrt{\Phi_{g}+\Phi_{h}}}=\frac{8}{\sqrt{2\times 2}+\sqrt{2+2}}+\frac{8}{\sqrt{3\times 2}+\sqrt{3+2}}\\ & \;+\frac{2}{\sqrt{3\times 3}+\sqrt{3+3}}=4.074 \end{array}$$

$$SRR\left(Naphazoline\right)=\underset{gh\varepsilon E\left(G\right)}{\sum }\frac{\Phi \left(h\right)-\Phi \left(l\right)+1}{\sqrt{\Phi \left(h\right)\times \Phi \left(l\right)}}=\frac{8\left(2-2+1\right)}{\sqrt{2\times 2}}+\frac{8\left(3-2+1\right)}{\sqrt{3\times 2}}+\frac{2\left(3-3+1\right)}{\sqrt{3\times 3}}=11.198$$

All the computations related to the 16 structures and 9 degree-based indices are done using the similar method given in Table 2.

**TABLE 2:** Calculated values of topological indices for eyes infection medicines.

Drug name	M_1_	M_2_	^*m*^ *M*_2_	H	F	HM	SD	FRR	SRR
Naphazoline	84	98	3.55	7.88	204	400	37.33	4.074	11.198
Tetrahydrozoline	80	95	3.33	7.4	196	521	35	3.355	7.449
Oxymetazoline	100	117	4.02	8.21	280	514	51.83	3.690	18.005
Pheniramine	86	96	4.13	8.56	206	398	41.66	4.433	13.529
Antazoline	100	113	4.52	9.83	236	462	45.66	5.067	13.776
Emedastine	112	131	5.02	10.6	276	538	51.33	5.490	16.328
Vidarabine	104	128	4.19	8.8	276	532	47.66	4.650	16.159
Foscarnet	30	30	1.5	2.48	96	156	21.50	1.407	10.041
Ciprofloxacin	128	155	5.02	10.83	336	646	59	5.740	19.926
Moxifloxacin	166	207	6.22	13.53	444	858	74	6.464	24.623
Gatifloxacin	150	185	5.86	12.4	402	772	69	6.590	23.889
Ofloxacin	146	180	5.52	11.83	394	754	67.66	6.326	23.390
Tobramycin	168	202	7.11	14.26	452	856	80	7.602	31.449
Amoxicillin	136	161	5.55	11.05	384	706	71.66	5.990	27.267
Dexamethasone	168	221	5.81	12.08	518	960	79.83	6.386	29.544
Chloramphenicol	94	106	4.61	8.8	244	456	49.66	4.655	19.855
Besifloxacin	150	184	5.77	12.33	402	770	69.66	6.576	23.890
Cyclopentolate	104	119	4.72	9.61	272	510	52	5.053	18.327
Nepafenac	94	108	4.27	8.73	238	454	46.33	4.586	16.160
Azithromycin	274	327	11.23	22.10	786	1440	142.25	11.985	52.669

## 4 Statistical computations

This section discusses the statistical calculations for the drugs used to treat eye infections. The computations are first conducted using the linear regression equation. Linear regression is used to calculate a linear relationship between two or more variables with normally distributed dispersion, homoscedasticity, no uncertainty in the predictors, variables used for the estimate, and independent observations. This section also covers the numerical values of the various statistical parameters, which are appropriate for examining the characteristics of anti-eye infection medications. The linear regression equation and parameters are computed for each topological index using Microsoft Excel. To study more information about correlation coefficient its validity, reliability and Objectivity see (Odom and MPEES, 2006; Koo et al., 2016).

### 4.1 Linear regression

Linear regression is a statistical method that utilizes a linear equation to establish the correlation between two variables. This technique is widely used in various fields, including finance, economics, and science, to predict future outcomes based on past data. The formula for linear regression is quite similar to the slope formula, and it is expressed as follows:

$$Y\left(Properties\right)=c+mX\left(TI\right),$$

where X is the independent variable and Y is the represented dependent variable. Topological indices are used to represent the independent values calculated by the molecular structures of drugs. These values are crucial in determining the dependent values for medications, which are their experimental values. The constant term c equals the value of Y when the value of X is 0, while the slope, or parameter m, shows how much Y varies for each unit change in X. To calculate the seven degree-based T-indices, a linear equation formula is used. The independent parameters used in this calculation include the first zagreb index, second zagreb index, modified second zagreb index, harmonic index, forgotten index, hyper zagreb index, symmetric division, first revised Randic, and second revised Randic indices. Each of these parameters has a different constant value and slope for various T-indices and physicochemical characteristics.

#### 4.1.1 First zagreb index M_1_(G)

$$\begin{array}{ll} & MW=19.3469+2.5028\left[M_{1}\left(G\right)\right]\\ & BP=332.6961+1.8512\left[M_{1}\left(G\right)\right]\\ & EV=51.0648+0.3116\left[M_{1}\left(G\right)\right]\\ & FP=155.0128+1.1196\left[M_{1}\left(G\right)\right]\\ & MR=8.5795+0.6296\left[M_{1}\left(G\right)\right]\\ & MV=4.0838+1.9676\left[M_{1}\left(G\right)\right] \end{array}$$

#### 4.1.2 Second zagreb index *M*_2_(G)

$$\begin{array}{ll} & MW=41.4333+1.9407\left[M_{2}\left(G\right)\right]\\ & BP=346.8257+1.4503\left[M_{2}\left(G\right)\right]\\ & EV=53.389+0.2445\left[M_{2}\left(G\right)\right]\\ & FP=163.5574+0.8771\left[M_{2}\left(G\right)\right]\\ & MR=14.7235+0.4842\left[M_{2}\left(G\right)\right]\\ & MV=24.8545+1.5026\left[M_{2}\left(G\right)\right] \end{array}$$

#### 4.1.3 Modified second zagreb index ^*m*^ *M*_2_(G)

MW=−15.3698+67.4754M2mGBP=311.1729+49.0934M2mGEV=47.4812+8.2571M2mGFP=141.996+29.6916M2mGMR=−1.4184+17.2208M2mGMV=−30.866+54.5462M2mG

#### 4.1.4 Harmonic index H(G)

$$\begin{array}{ll} & MW=-22.9778+33.2968\left[H\left(G\right)\right]\\ & BP=314.0578+23.4292\left[H\left(G\right)\right]\\ & EV=48.3294+3.9062\left[H\left(G\right)\right]\\ & FP=143.7425+14.1698\left[H\left(G\right)\right]\\ & MR=-4.3772+8.5941\left[H\left(G\right)\right]\\ & MV=-37.5078+26.9631\left[H\left(G\right)\right] \end{array}$$

#### 4.1.5 Forgotten index F(G)

$$\begin{array}{ll} & MW=53.9414+0.8281\left[F\left(G\right)\right]\\ & BP=353.5395+0.6268\left[F\left(G\right)\right]\\ & EV=54.0745+0.107\left[F\left(G\right)\right]\\ & FP=167.6163+0.3791\left[F\left(G\right)\right]\\ & MR=18.3298+0.2051\left[F\left(G\right)\right]\\ & MV=33.2354+0.6451\left[F\left(G\right)\right] \end{array}$$

#### 4.1.6 Hyper zagreb index HM(G)

$$\begin{array}{ll} & MW=42.1859+0.4515\left[HM\left(G\right)\right]\\ & BP=350.242+0.3329\left[HM\left(G\right)\right]\\ & EV=53.6718+0.05659\left[HM\left(G\right)\right]\\ & FP=165.6218+0.2013\left[HM\left(G\right)\right]\\ & MR=14.7955+0.1128\left[HM\left(G\right)\right]\\ & MV=23.5897+0.3525\left[HM\left(G\right)\right] \end{array}$$

#### 4.1.7 Symmetric division index SD(G)

$$\begin{array}{ll} & MW=26.5899+5.0684\left[SD\left(G\right)\right]\\ & BP=333.2125+3.8299\left[SD\left(G\right)\right]\\ & EV=50.7708+0.6512\left[SD\left(G\right)\right]\\ & FP=155.3241+2.3164\left[SD\left(G\right)\right]\\ & MR=10.9248+1.2661\left[SD\left(G\right)\right]\\ & MV=7.3944+4.0245\left[SD\left(G\right)\right] \end{array}$$

#### 4.1.8 First revised randic index FRR(G)

$$\begin{array}{ll} & MW=-4.9782+60.6484\left[FRR\left(G\right)\right]\\ & BP=317.1251+44.4185\left[FRR\left(G\right)\right]\\ & EV=48.8344+7.4068\left[FRR\left(G\right)\right]\\ & FP=145.598+26.8638\left[FRR\left(G\right)\right]\\ & MR=1.3378+15.4595\left[FRR\left(G\right)\right]\\ & MV=-19.3618+48.4636\left[FRR\left(G\right)\right] \end{array}$$

#### 4.1.9 Second revised randic index SRR(G)

$$\begin{array}{ll} & MW=64.1295+12.6864\left[SRR\left(G\right)\right]\\ & BP=343.9008+10.4334\left[SRR\left(G\right)\right]\\ & EV=52.3338+1.786\left[SRR\left(G\right)\right]\\ & FP=161.7866+6.3103\left[SRR\left(G\right)\right]\\ & MR=22.4465+3.0665\left[SRR\left(G\right)\right]\\ & MV=43.2706+9.7826\left[SRR\left(G\right)\right] \end{array}$$

### 4.2 Statistical parameters

This section includes information about all the statistical parameters, their significance, importance, and variations according to the T-indices. Seven parameters are used for the calculation of results. The term “N″ stands for the population of the sample. The value of N is 20 because 20 medicinal structures are used for computations. The parameter c is constant, and m is the slope of the linear regression. The term r is known as the Pearson correlation coefficient. The correlation coefficient is a particular measure that determines the strength of the linear relationship between two variables. It expresses the “degree of association” between two variables using just one number. The correlation lies between −1 and +1. Correlation coefficients that are negative show that when one variable increases, the other variable decreases. Positive correlation coefficients show that as one variable increases, the other increases as well. All the correlation coefficients have a numerical value above 0.6, which represents a good correlation. It is clear from Table 3–11 that the range of the correlation in the whole article is 0.6685–0.991. The correlation values between topological indices and the characteristics of the eye infection medicines are all positive and describe a direct relationship. In a regression model, R-Squared (also known as *r* ^2^ or the coefficient of determination) is a statistical measure that determines how much of the variation in the dependent variable can be explained by the independent variable. The parameter *r* ^2^ converts negative correlation into positive values, allowing it to be free of inverse and direct relationships. It is the absolute value of r. An element of the F distribution is the F value. The value can be used to evaluate the statistical significance of the test. Both the F-value and *p*-value are used to check the validity of the computations in an experiment. A *p*-value greater than 0.05 is not statistically significant and implies strong support for the null hypothesis. So the *p*-value must be greater than 0.05 for beneficial, evident, and useful outcomes. The data in Table 3–11 make it simple to see that all significant values of p are greater than 0.05.

**TABLE 3:** Computations of statistical data for *M*_1_(*G*).

Properties	N	c	m	r	*r* ^2^	F	p	Indicator
Molar Weight	20	19.3469	2.5028	0.9775	0.9555	386.7649	0.0000	Significant
Boiling Point	20	332.6961	1.8512	0.7038	0.4953	17.6655	0.0005	Significant
Enthalpy	20	51.0648	0.3116	0.7435	0.5528	22.254	0.00017	Significant
Flash Point	20	155.012	1.1196	0.7038	0.4953	17.667	0.00053	Significant
Molar Refraction	20	8.5795	0.6296	0.9573	0.9163	197.125	0.0000	Significant
Molar Volume	20	4.0838	1.9676	0.9136	0.8346	90.827	0.0000	Significant

**TABLE 4:** Computations of statistical data for *M*_2_(*G*).

Properties	N	c	m	r	*r* ^2^	F	p	Indicator
Molar Weight	20	41.4333	1.9407	0.9596	0.9209	209.6343	0.0000	Significant
Boiling Point	20	346.8257	1.4503	0.6981	0.4873	17.110	0.00061	Significant
Enthalpy	20	53.389	0.2445	0.7386	0.5456	21.610	0.0001	Significant
Flash Point	20	163.5574	0.8771	0.6981	0.4874	17.112	0.0006	Significant
Molar Refraction	20	14.7235	0.4842	0.9321	0.8688	119.227	0.0000	Significant
Molar Volume	20	24.8545	1.5026	0.8833	0.7803	63.9317	0.0000	Significant

**TABLE 5:** Computations of statistical data for ^*m*^ *M*_2_(*G*).

Properties	N	c	m	r	*r* ^2^	F	p	Indicator
Molar Weight	20	−15.3698	67.4754	0.991	0.9822	992.3369	0.0000	Significant
Boiling Point	20	311.1729	49.0934	0.7019	0.4927	17.4785	0.0005	Significant
Enthalpy	20	47.4812	8.2571	0.7408	0.5488	21.8964	0.0001	Significant
Flash Point	20	141.996	29.6916	0.7019	0.4927	17.4804	0.0005	Significant
Molar Refraction	20	−1.4184	17.2208	0.9847	0.9696	574.285	0.0000	Significant
Molar Volume	20	−30.866	54.5462	0.9524	0.9071	175.768	0.0000	Significant

**TABLE 6:** Computations of statistical data for H(G).

Properties	N	c	m	r	*r* ^2^	F	p	Indicator
Molar Weight	20	−22.9778	33.2968	0.976	0.9526	361.7849	0.0000	Significant
Boiling Point	20	314.0578	23.4292	0.6685	0.4469	14.5441	0.0012	Significant
Enthalpy	20	48.3294	3.9062	0.6994	0.4892	17.2408	0.0005	Significant
Flash Point	20	143.7425	14.1698	0.6685	0.4469	14.5449	0.0012	Significant
Molar Refraction	20	−4.3772	8.5941	0.9807	0.9618	453.554	0.0000	Significant
Molar Volume	20	−37.5078	26.9631	0.9396	0.8828	135.623	0.0000	Significant

**TABLE 7:** Computations of statistical data for F(G).

Properties	N	c	m	r	*r* ^2^	F	p	Indicator
Molar Weight	20	53.9414	0.8281	0.9683	0.9376	270.4136	0.0000	Significant
Boiling Point	20	353.5395	0.6268	0.7134	0.5089	18.6559	0.0004	Significant
Enthalpy	20	54.0754	0.107	0.7644	0.5844	25.3069	0.0000	Significant
Flash Point	20	167.6163	0.3791	0.7134	0.509	18.6597	0.0004	Significant
Molar Refraction	20	18.3298	0.2051	0.9338	0.8721	122.707	0.0000	Significant
Molar Volume	20	33.2354	0.6451	0.8968	0.8042	73.9228	0.0000	Significant

**TABLE 8:** Computations of statistical data for HM(G).

Properties	N	c	m	r	*r* ^2^	F	p	Indicator
Molar Weight	20	42.1859	0.4515	0.9564	0.9147	192.9692	0.0000	Significant
Boiling Point	20	350.242	0.3329	0.6865	0.4712	16.0406	0.0008	Significant
Enthalpy	20	53.6718	0.05659	0.7323	0.5362	20.8139	0.0002	Significant
Flash Point	20	165.6218	0.2013	0.6865	0.4713	16.0437	0.0008	Significant
Molar Refraction	20	14.7955	0.1128	0.9304	0.8657	116.061	0.0000	Significant
Molar Volume	20	23.5897	0.3525	0.8877	0.788	66.8911	0.0000	Significant

**TABLE 9:** Computations of statistical data for SD(G).

Properties	N	c	m	r	*r* ^2^	F	p	Indicator
Molar Weight	20	26.5899	5.0684	0.9888	0.9777	788.8539	0.0000	Significant
Boiling Point	20	333.2125	3.8299	0.7273	0.529	20.2147	0.0002	Significant
Enthalpy	20	50.7708	0.6512	0.776	0.6022	27.2452	0.0000	Significant
Flash Point	20	155.3241	2.3164	0.7273	0.529	20.2178	0.0002	Significant
Molar Refraction	20	10.9248	1.2661	0.9691	0.9247	221.107	0.0000	Significant
Molar Volume	20	7.3944	4.0245	0.9334	0.8712	121.717	0.0000	Significant

**TABLE 10:** Computations of statistical data for FRR(G).

Properties	N	c	m	r	*r* ^2^	F	p	Indicator
Molar Weight	20	−4.9782	60.6484	0.9805	0.9614	448.4644	0.0000	Significant
Boiling Point	20	317.1251	44.4185	0.699	0.4886	17.2001	0.0006	Significant
Enthalpy	20	48.8344	7.4068	0.7315	0.5351	20.7164	0.0002	Significant
Flash Point	20	145.598	26.8638	0.699	0.4887	17.201	0.0006	Significant
Molar Refraction	20	1.3378	15.4595	0.973	0.9468	320.2482	0.0000	Significant
Molar Volume	20	−19.3618	48.4636	0.9315	0.8676	117.9718	0.0000	Significant

**TABLE 11:** Computations of statistical data for SRR(G).

Properties	N	c	m	r	*r* ^2^	F	p	Indicator
Molar Weight	20	64.1295	12.6864	0.9756	0.9518	355.1799	0.0000	Significant
Boiling Point	20	343.9008	10.4334	0.781	0.61	28.1488	0.0000	Significant
Enthalpy	20	52.3338	1.786	0.839	0.7039	42.7939	0.0000	Significant
Flash Point	20	161.7866	6.3103	0.781	0.61	28.1561	0.0000	Significant
Molar Refraction	20	22.4465	3.0665	0.918	0.8428	96.5004	0.0000	Significant
Molar Volume	20	43.2706	9.7826	0.8943	0.7998	71.9198	0.0000	Significant

## 5 Graphical comparison of correlation coefficients

**FIGURE 3:**
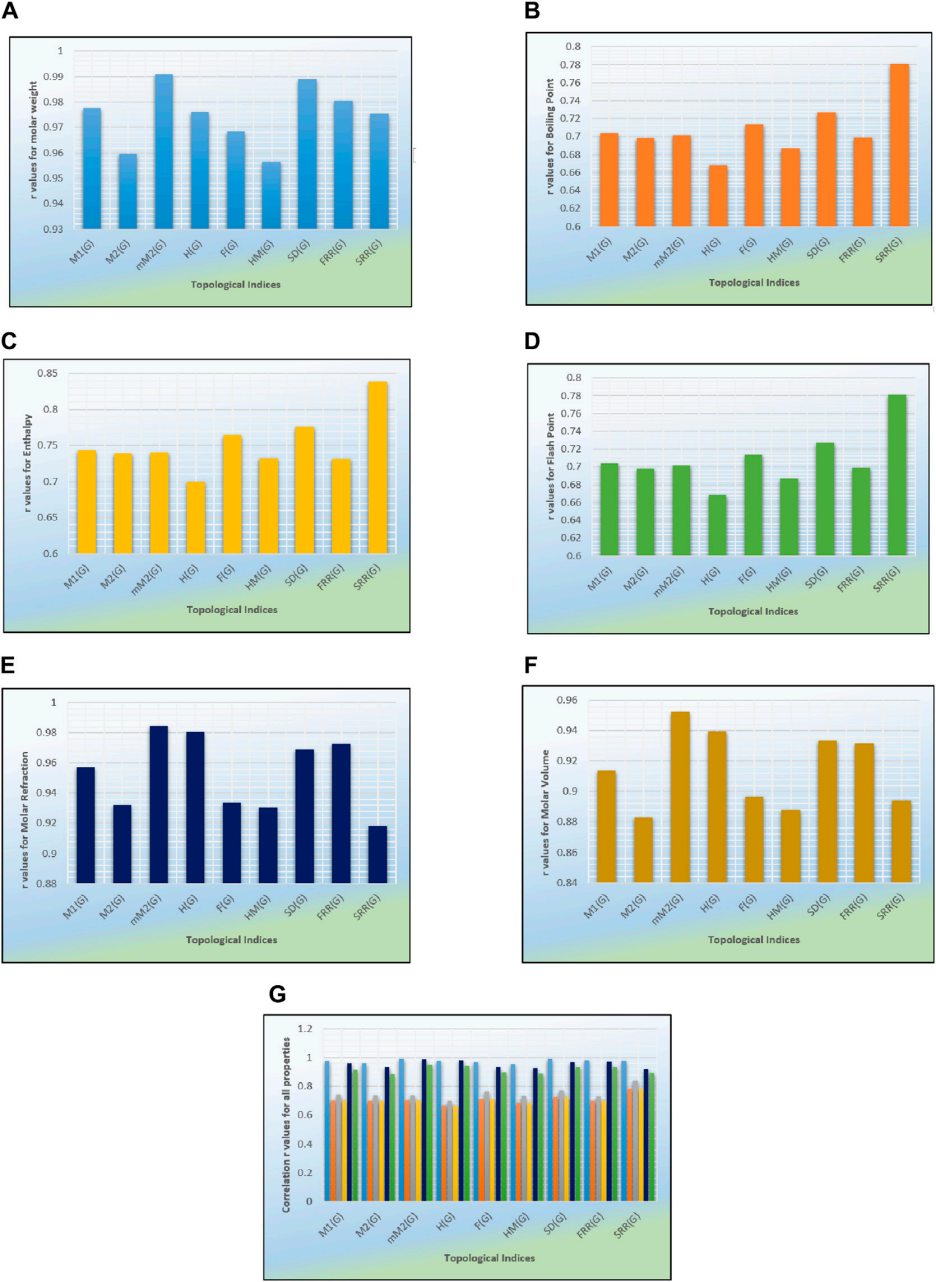
Graphical comparison of calculations.

Graphs are an essential tool for visually representing complex data relationships. They provide a concise and effective way to summarize information, making it more presentable and easier to understand. Graphs also facilitate comparisons between different sets of data, allowing for more informed decision-making. In Figure 3, a series of bar graphs illustrate the relationship between topological indices and the characteristics of anti-eye infection medicines. Each graph uses different colors to represent the varying correlations of the T-indices. For example, orange indicates the correlation of boiling points, which range from 0.6865 to 0.8. The *x*-axis displays the values of the topological indices, while the *y*-axis shows the values of correlation. The molar weight is a property whose highest correlation range lies between 0.95 and 1. The yellow-colored graph in Figure 3 represents the correlation between enthalpy and T-indices, which lies between 0.6994 and 0.839, indicating a good correlation. Line graphs and scatter graphs can also be useful for representing correlation points. The graph of the molar refractivity is depicted in dark blue and shows a strong and significant correlation of 0.9–1. The correlation of the flash point is indicated in green and lies between 0.6685 and 0.8. The brown-colored correlation graph between molar volume and degree-related T-indices shows an impressive range of results from 0.8 to 1. Notably, no correlation lies between 0 and 0.5, indicating a weak correlation.

Several articles have highlighted the importance of degree-related topological indices and linear regression in the field of medicine. In Table 12, we provide a comparison of different medicines and their corresponding outcomes. It can be observed that the correlation coefficients of the drugs used for eye infections are excessively high and suitable for use as an alternative to experimental values of drugs. As a result, we compare the results of malaria and cardiovascular drugs with those of eye infection drugs. Although the physicochemical properties of the medicines for eye infections have already been determined by laboratory experiments, this manuscript gives a theoretical way to estimate these properties using mathematical and statistical techniques.

**TABLE 12:** Comparison of results with literature.

Indices	Boiling point	Enthalpy of vaporization	Molar volume	Flash point
Results of the eye-infection medicines				
*M*_1_(*G*)	0.7038	0.7435	0.9136	0.7038
*M*_2_(*G*)	0.6981	0.7386	0.8833	0.6981
H(G)	0.6685	0.6994	0.9396	0.6685
(Bashir et al., 2022)Results of cardiovascular disease medicines				
*M*_1_(*G*)	0.814	0.778	0.975	0.809
*M*_2_(*G*)	0.840	0.811	0.959	0.835
H(G)	0.852	0.818	0.969	0.849
(Zhang et al., 2023)Results of malaria medicines				
*M*_1_(*G*)	0.961	0.968	0.735	0.961
*M*_2_(*G*)	0.962	0.978	0.645	0.963
H(G)	0.908	0.894	0.891	0.908

## 6 Applications of molecular descriptors

The Zagreb index is an important graph parameter that has extensive use in molecular chemistry, spectral graph theory, network theory, and various areas of mathematics and chemistry. It is a powerful tool that researchers employ to get information about the structure and properties of molecules. The M-indices were introduced for the computation of the pi-electron energies of chemical graphs. This manuscript discusses the six properties of eye infections. The MW, MR and MV can be predict by using second modified M-index with the values of correlation 0.991, 0.9847 and 0.9524 respectively. The BP (0.781), EV (0.839) and FP (0.781) has highest value of correlation with SRR-index, so SRR index is most suitable for prediction of these properties. The first Zagreb index is used to estimate the molar volume (0.9136) and molar refractivity (0.9573) because its correlation is near its maximum value. While other indices also have a significant correlation, the indices with the highest correlation are more accurate and suitable for computing the characteristics of anti-eye infection drugs. Therefore, researchers can use these indices to gain a better understanding of the properties of molecules and develop more effective drugs to combat eye infections. The interested reader might consult (Lee et al., 2022b; Mahboob et al., 2022) for more interesting applications of topological indices.

## 7 Discussion

The structures of the anti-eye infection drugs are examined and analyzed in this article by using seven degree-based topological indices. The characteristics of each novel drug’s structure must be known in order to produce it, and these characteristics can be uncovered through QSPR modeling using topological indices. The aim of this study is to apply topological indices to quickly and cost-effectively obtain information about the topology of a structure. Table 3–11 demonstrate the correlation coefficient between topological indices and the six physicochemical characteristics of the eye infection medications, providing valuable insight into the structure of these drugs. Through examination of the information presented in Table 3–11, it is possible to draw conclusions regarding the degree-related topological indices.

• The quantitative structure-property relationship (QSPR) study of the first M-index has revealed that the *M*_1_ index is a valuable tool for predicting the molar refractivity of drugs used to treat eye infections. The study found a strong correlation coefficient value of r = 0.9573, indicating that the *M*_1_ index is a reliable and accurate predictor of molar refractivity. This makes it a valuable resource for researchers in the field. The first M-index has a correlation range of 0.7–1 with various properties related to eye infection drugs, including MW (0.9775), BP (0.7038), EV (0.7435), FP (0.7038), MR (0.9573), and MV (0.9136). The p-values associated with the first M-index are also significant, further supporting its reliability. In comparison, the second M-index has a maximum correlation value of 0.9775 and a minimum value of 0.698. This information provides valuable insights into the predictive capabilities of the M-index in relation to molar refractivity and other properties of eye infection drugs.
• The modified second Zagreb index is the most suitable index for analyzing medicines used to treat eye infections because it has the highest correlation with three characteristics. It has the strongest association with molar weight (0.991), molar refractivity (0.9847) and molar volume (0.9524). Because no correlation value is negative, this index has a direct relationship with all of the physicochemical parameters of the eye infection structure. The most highest correlation in this paper is shown by ^*m*^ *M*_2_ index *r* = 0.991*≅*1.
• The QSPR investigation of the forgotten index has revealed that the F-index holds equal predictive power for the physical properties of eye infection treatments as the HM-index. The correlation coefficient falls within the range of 0.7134–0.9683, indicating a strong relationship. Particularly noteworthy is the robust correlation between the F-index and the molar weight of eye infection drugs, with a correlation value of 0.9683. As a result, the F-index has been discovered to be a useful tool in predicting the physical properties of eye infection drugs.
• The harmonic index and hyper-Zagreb index exhibit the strongest correlation values with molar refractivity and molar weight, standing at 0.9807 and 0.9683, respectively.
• The QSPR analysis presented in Table 9 demonstrates that the symmetric division index shows exceptional predictive capabilities in comparison to other degree-based topological indices. The correlation coefficients of the symmetric index with the attributes of eye infection medications range from 0.7273 to 0.9888, indicating a strong relationship. Particularly noteworthy are the correlation values of the symmetric index with three key properties: molecular weight (0.9888), molar refractivity (0.9691), and molar volume (0.9334). Additionally, all topological indices display a significant correlation exceeding 0.9 with molar weight, further underscoring the significance of the symmetric division index in predicting the characteristics of eye infection medicines.
• The newly introduced second revised Randic index has demonstrated its effectiveness as a predictive index for the properties of BP (0.781), EV (0.839), and FP (0.781). The correlation range for the SRR index falls between 0.781 and 0.9756. The strong correlation values with these three properties highlight the suitability of this index for analyzing drugs used in treating eye infections.
• The recently introduced first revised Randic index has been utilized to analyze the six characteristics of eye infection drugs. Upon reviewing Table 10, it is evident that the FRR index shows a correlation exceeding 0.9 for three characteristics: molar weight (0.9805), molar refractivity (0.973), and molar volume (0.9315). The p-values indicate the significance of the findings, with all p-values for the TIs indices being less than 0.001.

This study explores the use of degree-based T-indices in pharmaceutical for treating eye infections, which can be caused by lens wear, bacteria, fungi, viruses, or dust. The findings could help chemists and researchers in the pharmaceutical industry develop innovative drugs using these indices. The quantitative structure-property relationship (QSPR) analysis on 9 degree-based topological indices and 20 eye infection structures identified relevant indices with correlation coefficients ranging from 0.7 to 0.991. All models not only prove to be statistically significant but also demonstrate the best fit for the data. These results provide a solid foundation for the development of new drugs with similar structures, ultimately leading to enhanced efficacy and treatment outcomes. Some properties, such as the vapor pressure of the eye infection drugs, did not show a good correlation with the topological indices used in this paper, so we did not add these properties with a low correlation. Those properties and indices are discussed here, which show very good and significant correlation coefficients. The regression models that have been developed show strong correlation coefficients, which can greatly aid in the exploration of key features for designing novel drugs. However, certain characteristics of eye infection medications, such as density, pH values, index of refraction, and surface tension, cannot be accurately determined using these indices. This highlights the need for additional indices to thoroughly examine the chemical structures of various drugs, particularly those used in treating eye infections. Topological indices provide insights into the geometry, shape, and size of chemical structures, and their strong correlation with drug physical properties highlights their importance in pharmaceutical research and development.

## 8 Conclusion

In this paper, we utilize nine vertex degree-based topological indices to develop a quantitative structure-property relationship (QSPR) model for eye infection treatments. The model quantitatively correlates six properties of the drugs with nine topological indicators. Our linear regression model has been validated, confirming its predictive capabilities. This suggests that it could serve as a valuable tool for guiding drug development efforts in the medical field. The experimental data is taken from chemspider and required of numerical descriptors are obtained by by hand calculations and some Matlab tools. The values of numerical descriptor are actually the estimated values and its relationship with the experimental values are done by the help of statistical tools. All the values of correlation coefficients, *p*-values (less then 0.05) and F-statics (greater than 2.5) for all the data give significant outcomes.The QSPR analysis of the eye infection drugs shows that the molar refractivity of these drugs can be explained with the help of the first Zagreb index, with a correlation value of 0.9163. The molar weight has the strongest relationship with the modified second Zagreb index, so the molar weight of the eye infection drugs will be estimated by this index. The relationship between the symmetric division index and all six properties of the medicines is significant. It is clear from the tables of statistical data that two novel indices have also proved to be successful for prediction properties. Three properties, such as boiling point (0.781), enthalpy of vaporization (0.839), and flash point (0.781), can be predicted by the second revised Randic index. A large number of articles have been written on the same method to understand different chemical structures, but first-time eye infection drugs are studied with the help of degree-based indices. We compared the results of eye infection drugs with some papers in the literature with the same topological indices to show the validity of the results. 

This research has the potential to significantly impact the field of pharmaceutical sciences, offering valuable insights for extrapolating the physicochemical characteristics of novel drug designs to address various specific disorders. One of the main challenges is the availability of reliable and high-quality data, as constructing accurate models requires a large amount of experimental data. Additionally, interpreting the results of QSPR models can be complex due to their inherent nature, making it difficult to understand why certain predictions are made. Another difficulty is selecting appropriate molecular descriptors and mathematical algorithms for model development, as different methods may produce varying results. This limitation can result in low prediction accuracy, particularly for pharmaceutical with unconventional molecular structures.

### 8.1 Future work

In a similar way, we try to study the properties of other drugs with the help of different topological indices. To gain new insights into the different chemical structures, we can introduce new topological indices to explore them. This could open up a world of possibilities for future research.

## Data Availability

The original contributions presented in the study are included in the article/Supplementary material, further inquiries can be directed to the corresponding author.
